# The impact of self-transcendence values on cooperative behaviors: attitude mediation and emotional moderation

**DOI:** 10.3389/fpsyg.2025.1701352

**Published:** 2026-01-12

**Authors:** Qian-Qian Kong, Harmi Izzuan Bin Baharum

**Affiliations:** 1 Language Academy, Faculty of Social Sciences and Humanities, Universiti Teknologi Malaysia, Kuala Lumpur, Malaysia; 2 Department of Foreign Languages, Jining University, Jining, China

**Keywords:** cooperative attitudes, cooperative behaviors, cooperative learning, emotional intelligence, foreign language enjoyment, self-transcendence values

## Abstract

This study investigated the psychological mechanisms through which Self-transcendence values (ST), Emotional Intelligence (EI), and Foreign Language Enjoyment (FLE) influence cooperative behaviors among Chinese university-level English learners (*N* = 561). The results, analyzed using structural equation modeling (SEM), provided strong support for a “Value-Attitude-Behavior” (VAB) model. Specifically, ST was a significant positive predictor of cooperative attitudes, which in turn served as a key mediator in facilitating cooperative behaviors. Both EI and FLE were found to be significant positive contributors to cooperative attitudes and behaviors. A notable finding was that EI and FLE exhibited significant negative moderating effects on the relationship between ST and cooperative attitudes, indicating a non-linear, inhibitory role in the value transmission pathway. This study validates and extends the applicability of the VAB model to the domain of second language acquisition, highlighting the complex interplay between personal values and affective factors in shaping cooperative outcomes.

## 1 Introduction

In the context of the digital era, Cooperative Learning (CL) continues to gain significant attention from educational researchers and practitioners due to its crucial value in fostering communication skills, cooperative abilities, and problem-solving capabilities (Johnson and Johnson, 2019; Jacobs and Ivone, 2020). Extensive research confirms that CL not only promotes positive development in students' mental health and social relationships (Johnson and Johnson, 2009; Van Ryzin and Roseth, 2018; Tolmie et al., 2010; Lavasani and Khandan, 2011) but also significantly enhances their academic achievement (Yamarik, 2007; Suh, 2009; Zhang et al., 2021).

However, the application of CL faces challenges related to individual adaptability differences. For example, students who prefer independent thinking may struggle to concentrate during highly interactive CL sessions (Felder and Silverman, 1988), while others may exhibit low participation or even resistance in collaborative settings (Bonwell and Eison, 1991). As highlighted by Chen (2014), groups that fail to establish a dynamic equilibrium and effective “mutual promotion and restraint” among members often encounter difficulties in reaching consensus, engaging in in-depth discussions, or forming effective actions. These variations suggest that individual predispositions significantly influence how students engage with and benefit from CL. Although prior studies have acknowledged the role of such differences, the psychological mechanisms explaining why some students thrive in CL while others struggle remain inadequately explored.

To address this gap, this study adopts a motivational lens grounded in value theory. Values, particularly Self-Transcendence (ST) values—emphasizing universalism and benevolence—serve as fundamental motivators that predispose individuals toward cooperative behaviors. Research by Schwartz (1996) confirms that ST values significantly and positively predict cooperation. Subsequent studies further indicate that ST values is not only a key predictor of student engagement in CL (Bogaert et al., 2012; Pletzer et al., 2018), but also crucial for a group's innovative capacity (Choi and Yoon, 2018). Nevertheless, while the direct correlation between ST values and cooperation is acknowledged, the psychological pathways through which ST values translate into actual cooperative behaviors within CL contexts are not well understood, representing a critical theoretical ambiguity.

The Value–Attitude–Behavior (VAB) hierarchy provides a robust theoretical lens through which to examine the psychological pathway from values to action. As established by Homer and Kahle (1988), values shape mid-range attitudes, which in turn influence specific behaviors. In the context of CL, this model implies that ST values foster a positive Cooperative Attitudes (CA), which subsequently facilitates active Cooperative Behaviors (CB). This pathway may be particularly salient in collectivist cultural settings, such as among Chinese university students, where Confucian values emphasizing harmony and collective welfare may strengthen the link between ST values and CA.

However, the translation of values into consistent behaviors is not direct; it is mediated by both volitional strategies and emotional capacities. Independent learning skills—such as organization, time management, rehearsal, and self-regulation—serve as key volitional mechanisms through which values are operationalized into action. The activation of these strategies is itself shaped by the broader cultural context. For instance, a cross-cultural study comparing Hong Kong and UK students revealed that those from Hong Kong, influenced by educational traditions stressing discipline and diligence, tended to view independent learning primarily as efficient management of time and content. In contrast, British students exhibited stronger self-efficacy, critical thinking, and self-regulation (Lo et al., 2023). These differences underscore that the VAB pathway is culturally filtered, not universal.

Emotional factors further moderate this process. Emotional intelligence (EI), defined as the ability to perceive, use, understand, and manage emotions, supports the navigation of social interactions in CL. Individuals with high EI are better equipped to align their behaviors with ST values, thereby strengthening the coherence between ST values and cooperative conduct. Similarly, foreign language enjoyment (FLE)—a positive emotional state in language learning that broadens cognitive and behavioral repertoires—can enhance the link between ST values, CA and CB. Enjoyable collaborative experiences reinforce CA and make the transition to sustained cooperation more likely. Thus, the VAB framework, when integrated with cultural, strategic, and emotional mediators, offers a comprehensive model for understanding how values translate into CB.

This study seeks to advance a nuanced understanding of CL by examining a moderated mediation model. It investigates the pathway from ST values to CB via CA, while testing the moderating role of EI and FLE in the ST–CA relationship. This integrated approach aims to elucidate the motivational and affective mechanisms driving successful cooperation in language learning, offering insights particularly relevant to collectivism-oriented educational settings.

## 2 Literature review

### 2.1 Personal values and attitudes

Based on Schwartz's (2016) theory of universal values, the fundamental structure of human motivation comprises ten basic values arranged in a circular continuum, summarized by two polarized dimensions: self-transcendence vs. self-enhancement and openness to change vs. conservation. Personal values form the intrinsic justification for an individual's adherence to corresponding attitudes (Katz, 1960). Cross-cultural empirical studies have confirmed significant associations between values and attitudes (Boer and Fischer, 2013; Filippou et al., 2022): Boer and Fischer (2013) found that ST values significantly and positively predict individuals' prosocial attitudes. Filippou et al. (2022) revealed that pre-service teachers' identification with ST values significantly and positively predicts their positive attitudes toward CL. Integrating the theoretical framework and empirical evidence, this study proposes:

H1: A significant positive association exists between students' adherence to ST values and their cooperative attitudes (CA).

### 2.2 Personal values and behaviors

According to Schwartz's (1977) Norm Activation Theory, personal values guide specific behavioral choices by activating internal behavioral norms. Cross-cultural research by Bardi and Schwartz (2003) indicates significant differences in the strength of association between different types of values and their corresponding behaviors: ST exhibit the strongest correlation with altruistic behaviors, while values such as hedonism show only moderate levels of association with their corresponding behaviors. In dilemma experiments involving resource allocation, individuals with higher levels of ST values demonstrate a more pronounced tendency toward cooperation (Schwartz, 1996). Based on this theoretical framework and empirical evidence, this study proposes:

H2: A significant positive association exists between students' adherence to ST values and their cooperative behaviors (CB).

### 2.3 Value–attitude–behavior hierarchy

The Value–Attitude–Behavior Model (VAB Model), proposed by (Homer and Kahle 1988), reveals the mediating effect of attitudes between values and behaviors. This model has received cross-contextual empirical support across multiple domains, including education (Shim et al., 1999), public environmental decision-making (Vaske and Donnelly, 1999), and tourism management (Han et al., 2019). Focusing on the field of education, Tal and Yinon (2002) confirmed that value identification can influence teaching practices through the mediating role of attitudes. Integrating the core theoretical framework of the VAB Model and its empirical validation across different domains, this study proposes:

H3: Cooperative attitudes (CA) mediate the relationship between ST values and cooperative behaviors (CB).

### 2.4 Moderating mechanism of Emotional Intelligence (EI)

Emotional Intelligence (EI), conceptualized as the capacity to perceive, understand, utilize, and manage one's own and others' emotions, constitutes a fundamental human ability that significantly influences behavioral performance and subjective well-being (Mayer and Salovey, 1997). Its impact extends beyond individual functioning to broader organizational and educational contexts. For instance, a comprehensive analysis of 104 studies revealed that leaders with high EI not only optimize their own performance and business outcomes but also significantly enhance overall team effectiveness (Coronado-Maldonado and Benítez-Márquez, 2023). Within the specific domain of foreign language learning, EI has been demonstrated to effectively alleviate language anxiety and serve as a positive predictor of academic achievement (Dewaele et al., 2008; Pishghadam, 2009; Li, 2018, 2020).

Critically, while Emotional Intelligence (EI) and an individual's internalized value systems (e.g., ST values) are distinct personal factors that can operate independently, Bandura's (1986) theory of Triadic Reciprocal Determinism provides a lens to understand their dynamic interplay in influencing behavior. Within this framework, EI emerges as a key personal factor—a core capability that enables individuals to accurately recognize and understand their own and others' emotional states. This capacity is essential for navigating the social complexities inherent in CL environments (Goleman, 1995). By fostering improved group dynamics, empathy, and effective collaboration, high EI equips individuals to perform more effectively in cooperative settings, which in turn leads to enhanced academic outcomes and interpersonal relationships (Parker et al., 2004; Petrides et al., 2004).

From the perspective of Pekrun's (2006) Control-Value Theory, EI facilitates adaptive behavior through two core mechanisms: EI strengthens perceived control by managing social-interactive anxiety, thereby sustaining emotional stability during cooperative tasks; EI enhances value appraisal by aligning emotional experiences with prosocial goals (e.g., ST values), thus promoting value-driven attitudes. Together, these mechanisms reposition EI as a metacognitive resource that optimizes the antecedent conditions—control and value appraisals—for CB. Building on this integrated perspective, we propose that EI acts not merely as a direct predictor but as a moderator in the value–behavior sequence.

While EI bolsters the translation of ST values into cooperative outcomes by helping individuals manage socio-emotional challenges, its role may be nuanced. Drawing upon the Emotions as Social Information (EASI) theory, high-EI individuals exhibit heightened sensitivity to real-time emotional cues. This may lead them to prioritize immediate interpersonal signals—such as group members' anxiety or joy—over distal values when forming CA. This tendency is further reinforced in CL contexts, where richer and higher-quality feedback increases the demand for EI-related socio-emotional decoding. Consequently, the continuous processing of such nuanced emotional information may attenuate the influence of ST values on attitude formation, as EI enables individuals to adapt more flexibly to dynamic group interactions. As a result, the influence of ST values on CA may be attenuated when EI is high, as attitude formation becomes more contingent on the immediate social context. Thus, we hypothesize:

H4: A significant positive association exists between EI and Cooperative Attitudes (CA).

H5: A significant positive association exists between EI Cooperative Behaviors (CB).

H6: EI negatively moderates the relationship between ST values and Cooperative Attitudes (CA).

### 2.5 The moderating mechanism of Foreign Language Enjoyment (FLE)

With the in-depth development of positive psychology in the field of educational research, the impact of positive emotions on language acquisition has increasingly garnered academic attention (Dewaele and Proietti Ergün, 2020). Among these, Foreign Language Enjoyment (FLE) is widely regarded as a key variable predicting language learning outcomes (Oxford, 2014), owing to its ability to effectively expand an individual's cognitive resources (Fredrickson, 2001). A series of studies has confirmed that FLE significantly promotes learners' academic achievements (Botes et al., 2022; Fathi et al., 2023; MacIntyre and Gregersen, 2012; Dewaele et al., 2023; Dong and Liu, 2022; Ma et al., 2023; Cui and Meng, 2023). Zhang et al. (2023) pointed out that in English CL activities, positive emotions (centered around enjoyment, pride, and enthusiasm) accounted for 63.18% of the observed emotions, and these positive emotions enhanced the effectiveness of group interactions. FLE represents a positive emotional state wherein learners become deeply immersed during language practice activities, owing to a sense of task mastery and achievement orientation (Li et al., 2018). A meta-analysis of 56 studies conducted by Botes et al. (2022) demonstrated that FLE exhibits strong positive correlations with willingness to communicate (*r* = 0.48), moderate positive correlations with academic achievement (*r* = 0.30), and self-perceived achievement (*r* = 0.27). Dewaele et al. (2023) further highlighted that FLE, Foreign Language Classroom Anxiety (FLCA), and Foreign Language Boredom (FLB) all significantly predict academic achievement, with FLCA exerting the strongest influence, followed by FLE, and then FLB.

In the context of CL, Zhang et al. (2023) examined the effects of learners' emotions on their affective engagement in English CL settings. Their findings indicated that CL exhibit the richest emotion nodes, the highest network density, and the most frequent transitions. Additionally, Fathi et al. (2023) found that FLE, combined with the ideal EFL self and intercultural communicative competence, serves as a significant predictor of willingness to communicate in English as a foreign language.

According to Pekrun's control-value theory, students' sense of control over learning activities and their recognition of task value are key antecedents to academic emotions, which in turn influence engagement and outcomes. In this light, Lo (2023) emphasizes that intentionally designed learning experiences—such as those in CL that provide rich feedback, clear standards, and iterative improvement opportunities—can foster positive emotions and perceived control, thereby boosting self-efficacy and academic performance. However, not all emotional experiences uniformly facilitate motivation. Drawing on Deci's (1971) overjustification theory, if learners attribute enjoyable CL experiences primarily to external rewards rather than intrinsic value, their intrinsic motivation for cooperation may be undermined. Thus, while FLE generally supports CA and CB, it may also weaken the relationship between ST values and CA when enjoyment is externally attributed. Based on this reasoning, we hypothesize:

H7: A significant positive association exists between FLE and Cooperative Attitudes (CA).

H8: A significant positive association exists between FLE and Cooperative Behaviors (CA).

H9: FLE negatively moderates the relationship between ST values and Cooperative Attitudes (CA).

**Figure 1:**
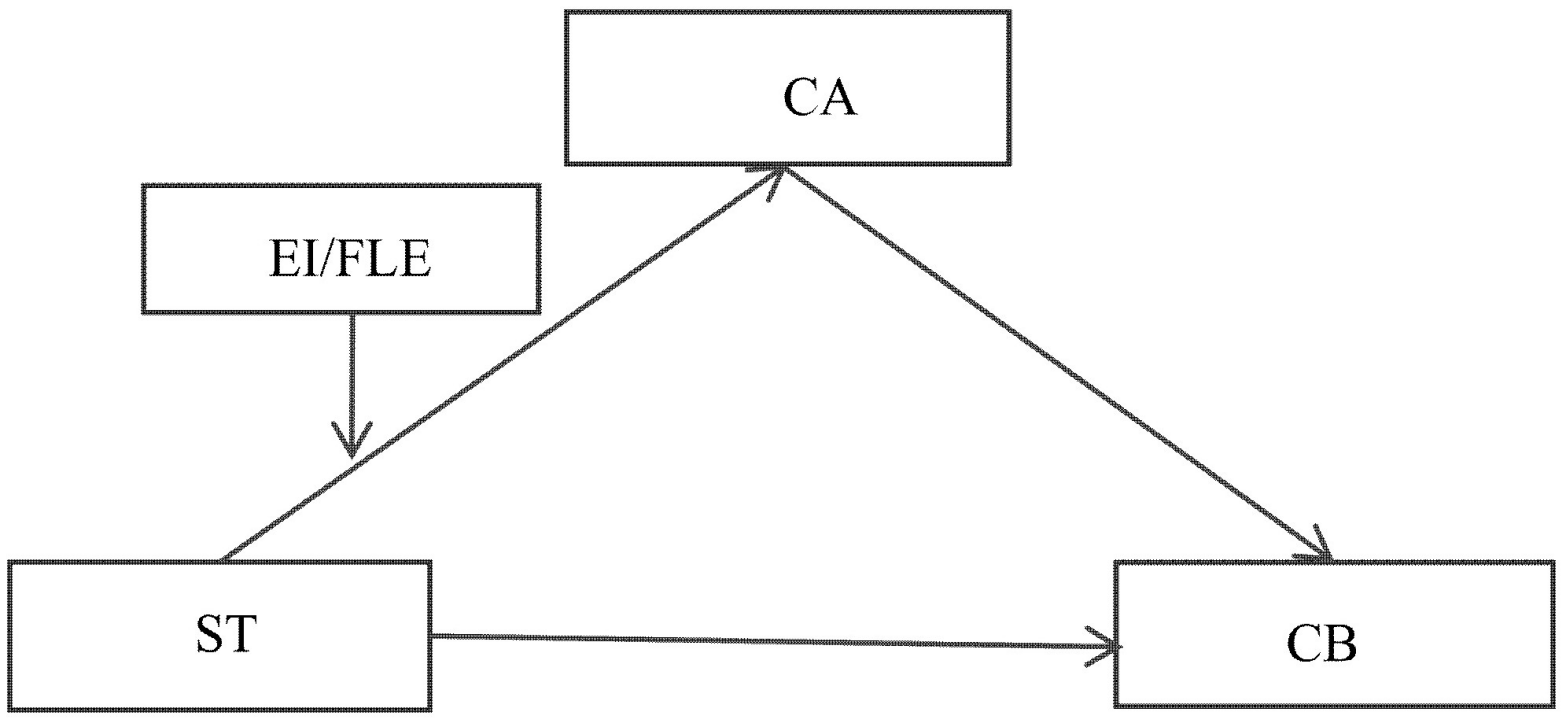
Research model.

Based on the above series of theoretical deductions and hypothesis construction, this study integrates five core constructs—ST values, CA, CB, EI, and FLE—to propose an overall theoretical framework revealing their intrinsic pathways of influence, as specifically illustrated in Figure 1.

## 3 Materials and methods

### 3.1 Research participants

Data were collected through the Wenjuanxing online survey platform utilizing a convenience sampling method. As a complete sampling frame of all eligible full-time undergraduates was unavailable, participants were recruited through established contacts with English instructors at six universities across three provinces. With the instructors' permission, questionnaires were distributed during or after English classes. The data collection procedure adhered to strict ethical standards. All participants were comprehensively briefed on the study's objectives, data usage, and confidentiality measures. The survey was conducted anonymously, with data used solely for academic purposes.

Prior to analysis, we implemented a data cleaning protocol. First, we examined missing values for all variables and confirmed that the final dataset used for analysis was complete, with no missing values. Subsequently, responses were screened against objective criteria to exclude invalid questionnaires, such as those exhibiting patterned responses (e.g., selecting the same option like “7” across all items). This process led to the exclusion of two such invalid questionnaires, resulting in a final valid sample of 561 participants for all subsequent analyses.

Table 1 shows the sample (*n* = 561) comprised predominantly female students (77.5%, *n* = 435) aged 18–24 years (*M* = 19.78, *SD* = 1.34). Most participants were enrolled in English-related majors (63.5%, *n* = 356), with the remainder from non-language disciplines (36.5%, *n* = 205). The participants were predominantly undergraduates (93.8%, *n* = 526); a minority held college diplomas (5.5%, *n* = 31) or vocational degrees (0.7%, *n* = 4). Nearly all participants (96.3%, *n* = 540) reported prior experience with CL.

**Table 1:** Demographic characteristics.

**Category**	**Item**	**Number**	**Percentage (%)**
Major	English	285	50.8
	Business English	71	12.7
	Non-language	205	36.5
Education level	Bachelor's degree	526	93.8
	Associate degree	31	5.5
	Vocational college	4	0.7
CL experience	With experience	540	96.3
	Without experience	21	3.7
Sex	Male	126	22.5
	Female	435	77.5

### 3.2 Research instruments

This study employed the following scales to measure the core constructs:

#### ESS21 Values Scale

The Chinese version of ESS21 Values Scale was revised by Chen Dadi based on several previous translations (Schwartz, 2016). The translation rigorously followed the iterative back-translation guidelines outlined by the European Social Survey (ESS) (Schwartz, 2016). This multi-step procedure involves independent translation, back-translation, review by the original author (Shalom Schwartz), and revisions until consensus is achieved, ensuring conceptual equivalence (Schwartz, 2016). The Chinese version of this scale was used to assess learners' personal value orientations. It consists of 21 items. To maintain consistency with other measurement tools, the original reverse 6-point scoring system was adapted to a 7-point Likert scale, ranging from 1 (“Completely unlike me”) to 7 (“Very much like me”). This adaptation was implemented to maintain internal consistency across all measurement tools in the study, thereby reducing cognitive load for respondents.

To evaluate the structural validity of the ST values subscale, which consists of five items, a confirmatory factor analysis (CFA) was conducted. The single-factor model exhibited an acceptable fit to the data: χ^2^/d*f* = 4.550, *p* < 0.001, CFI = 0.986, TLI = 0.971, RMSEA = 0.080 (90% CI: 0.048, 0.114). Although the significant chi-square statistic and elevated χ^2^/d*f* ratio may reflect sensitivity to the substantial sample size, the key relative fit indices (CFI and TLI) surpassed the 0.95 benchmark for excellent fit, and the RMSEA value met the acceptable criterion of < 0.08. These findings collectively support the good fit of the measurement model, indicating satisfactory structural validity of the ST subscale in this study. Additionally, psychometric analyses confirmed that the adapted 7-point Likert scale maintained excellent internal consistency (Cronbach's α = 0.861).

The varying treatments of the ST values variable across analyses were guided by methodological requirements and theoretical considerations. In descriptive statistics, ST scores were intra-individually centered (MRAT) to control for individual response biases, transforming them into relative importance scores within each participant's value system. For structural equation modeling (SEM), ST values were modeled as a latent variable defined by its five item indicators, which allows the model to account for measurement error and obtain a more robust representation of the underlying construct. In the moderation analysis, a standardized composite score of ST values was used to ensure all variables were on a comparable metric, as scaling inconsistencies can distort interaction terms and complicate the interpretation of moderation effects.

#### Trait Emotional Intelligence Questionnaire-Short Form (TEIQue-SF)

EI was measured using the trait scale developed by Petrides (2009). We employed the Chinese version revised by Li (2018). The translation and cross-cultural adaptation of this version were conducted by two bilingual experts in psychology and linguistics (Li, 2018). Subsequent validation studies confirmed its satisfactory reliability and validity (e.g., Li, 2018; Li et al., 2018; Li, 2020). This scale comprises 30 items rated on a 7-point Likert scale (1 = “Strongly disagree”, 7 = “Strongly agree”). It includes 15 reverse-scored items (Items 2, 4, 5, 7, 8, 10, 12, 13, 14, 16, 18, 22, 25, 26, 28), which were reverse-coded prior to data analysis.

#### Foreign Language Enjoyment Scale

FLE was measured based on the Foreign Language Enjoyment Scale (FLES) by Dewaele and MacIntyre (2014). Similarly, we adopted the Chinese version revised by Li (2018). The translation process similarly involved rigorous procedures, including forward-translation, back-translation, and review by bilingual experts. Subsequent validation studies confirmed its satisfactory reliability and validity (e.g., Li, 2018; Li et al., 2018; Li, 2020). This version contains 11 items, also rated on a 7-point Likert scale (1 = “Strongly disagree”, 7 = “Strongly agree”).

#### Cooperative Attitudes Scale

CA was measured using a self-developed scale based on Shavitt's (1990) value-attitude theoretical framework. An initial pool of seven items was generated and pilot-tested with a sample of 234 participants. Following item analysis, two items with weak psychometric properties (e.g., low factor loadings) were removed, resulting in a final 5-item scale. All items are rated on a 7-point Likert scale (1 = “strongly disagree” to 7 = “strongly agree”). The final items are as follows:

a) Personal development and progress cannot be separated from cooperation between people.
b) I think working with others can help us learn from each other and make progress together.
c) I believe that only good cooperation can lead to development.
d) I think cooperation is everywhere.
e) It is essential for everyone to cooperate with others.

#### Cooperative Behaviors Scale

The measurement of CB adopted the Chinese revised version of the “Cooperative Learning Behavior Scale” by (Fernandez-Rio et al. 2021). This scale includes 15 items, covering five dimensions: goal interdependence, interpersonal interaction, personal responsibility, group reflection, and communication skills. It uses a 7-point Likert scoring system (1 = “strongly disagree”, 7 = “strongly agree”).

The English version of the CA and CB scale was translated into Chinese following a rigorous translation and back-translation procedure. Two independent bilingual experts performed the translation and back-translation, and the final version was reviewed and approved by the authors to ensure linguistic and conceptual accuracy.

The reliability indicators (Cronbach's α coefficients) for all scales in this study were good, specifically: ESS21 Values Scale 0.861, TEIQue-SF.902, Foreign Language Enjoyment Scale 0.929, Cooperative Attitudes Scale 0.952, and Cooperative Behaviors Scale 0.983.

### 3.3 Data analysis

The data analysis followed a sequential approach, beginning with preliminary assessments and exploratory factor analysis (EFA), followed by confirmatory factor analysis (CFA) and hypothesis testing using structural equation modeling (SEM) and regression-based analyses. The specific procedures are detailed below.

To mitigate the potential for common method bias (CMB), several procedural remedies were incorporated into the survey design, including spatial separation (multi-institution sampling), temporal separation (questionnaires distributed in batches), and respondent anonymity (Podsakoff et al., 2003). Prior to the main analysis, the suitability of the data for factor analysis was confirmed. The Kaiser-Meyer-Olkin (KMO) measure was 0.963, well above the recommended threshold of 0.6 (Kaiser and Rice, 1974), and Bartlett's Test of Sphericity was significant (χ^2^ = 39808.76, *p* < 0.001), supporting the factorability of the correlation matrix (Hair et al., 2019). An Exploratory Factor Analysis (EFA) using principal component analysis was then conducted. The analysis extracted 13 factors with eigenvalues greater 1, collectively accounting for 69.075% of the total variance. Of note, the first factor explained 35.199% of the variance, which is below the 40% threshold, suggesting that serious common method bias was not a major concern in the data.

To ensure the data met the fundamental distributional assumptions for structural equation modeling (SEM), a comprehensive normality assessment was conducted. The evaluation of univariate normality, based on skewness and kurtosis, indicated that all variables were within acceptable ranges (absolute skewness < 0.73, absolute kurtosis < 0.67), well below the conservative thresholds of 3 and 10 (Kline, 2016), respectively. However, Mardia's (1970) test revealed a significant deviation from multivariate normality (coefficient = 147.762, c.r. > 1.96). Consequently, to ensure robust parameter estimates, the maximum likelihood estimation method with robust corrections (MLR) was employed, which provides Satorra–Bentler scaled chi-square values and standard errors that are resilient to non-normal data.

On this basis, the study proceeded with the following analyses. The convergent validity of the measurement model was evaluated using Composite Reliability (CR) and Average Variance Extracted (AVE) based on the standards proposed by Hair et al. (2019) with thresholds of CR ≥ 0.7 and AVE ≥ 0.5. Discriminant validity was assessed using the Fornell–Larcker criterion (1981). Multicollinearity issues were examined through Tolerance, Variance Inflation Factor (VIF), and Eigenvalues. The foundational characteristics of the data were analyzed using descriptive statistics (mean and standard deviation). Confirmatory Factor Analysis (CFA) was conducted using AMOS 26 software, with model fit evaluated via the maximum likelihood estimation method. A path model of “ST → CA → CB” was constructed based on AMOS 26, and the significance of indirect effects was tested using the bias-corrected Bootstrap method (with 5,000 repeated samples and a 95% confidence interval). The moderating effects of EI and FLE were analyzed using the PROCESS macro in SPSS 26, based on the principles of hierarchical regression.

## 4 Results

### 4.1 Measurement model testing

The confirmatory factor analysis (CFA) results for the five-factor structure comprising ST values, CA, CB, EI, and FLE indicated that the model fit indices met acceptable levels: χ^2^/d*f* = 3.224, CFI = 0.966, TLI = 0.960, RMSEA = 0.063, RMR = 0.059. To assess the potential influence of common method bias (CMB), we employed the unmeasured latent method factor (ULMF) approach for statistical control (Podsakoff et al., 2003). Two nested CFA models were compared: a baseline model (containing only the five theoretical constructs) and an extended model that included an additional common method factor influencing all measurement items, with its paths constrained to be equal and variance fixed to 1. The results showed that the model fit of the extended model (χ^2^/d*f* = 3.240, CFI = 0.965, TLI = 0.960, RMSEA = 0.063) was highly similar to that of the baseline model, with no significant improvement (ΔCFI = 0.001). According to the conventional criterion (ΔCFI < 0.01; Cheung and Rensvold, 2002), common method bias is not a serious concern in this study, thereby supporting the robustness of the subsequent analyses.

As shown in Table 2, the *CR* values ranged from 0.785 to 0.984, all exceeding the threshold criterion (*CR* ≥ 0.7) recommended by Fornell and Larcker (1981), demonstrating excellent internal consistency of the scales. The *AVE* values ranged from 0.550 to 0.927, all surpassing the minimum standard (*AVE* ≥ 0.5) set by Gefen and Straub (2005), confirming that the convergent validity of each latent variable was achieved.

**Table 2:** Convergent validity.

**Item**	**Std**.	**UnStd**.	**S.E**.	**C.R**.	***p***	**CR**	**AVE**
EI	0.803	1				0.885	0.657
	0.786	0.846	0.043	19.898	^***^		
	0.819	0.724	0.035	20.913	^***^		
	0.834	0.866	0.041	21.354	^***^		
ST	0.709	1				0.863	0.560
	0.799	1.004	0.057	17.687	^***^		
	0.816	1.048	0.058	18.048	^***^		
	0.784	1.041	0.06	17.374	^***^		
	0.613	0.895	0.065	13.683	^***^		
CA	0.933	1				0.954	0.806
	0.911	0.941	0.024	38.607	^***^		
	0.949	0.959	0.021	44.706	^***^		
	0.796	0.931	0.034	27.106	^***^		
	0.894	0.964	0.026	36.398	^***^		
CB	0.957	1				0.984	0.927
	0.972	1.008	0.017	60.513	^***^		
	0.956	0.988	0.018	54.455	^***^		
	0.973	1.012	0.017	60.801	^***^		
	0.956	1.016	0.019	54.267	^***^		
FLE	0.7	1				0.785	0.550
	0.722	0.845	0.058	14.502	^***^		
	0.8	1.193	0.077	15.455	^***^		

^***^p < 0.001.

The discriminant validity among the five latent variables is shown in Table 3. Initial assessment using the Fornell-Larcker criterion indicated a potential issue, as the correlation coefficients between ST values and CA (0.764) and between ST values and CB (0.797) exceeded the square root of the AVE for ST (0.748). To address this more rigorously, we employed supplementary analyses. First, although the HTMT values (ST-CA: 0.744; ST-CB: 0.779) were below the 0.85 threshold (Henseler et al., 2015), a bootstrap procedure with 5,000 samples was conducted to estimate bias-corrected 95% confidence intervals (CIs) for the latent variable correlations. The results showed that the upper bounds of all CIs were substantially below 1.0 (e.g., ST-CA: [0.697, 0.822]; ST-CB: [0.741, 0.847]), allowing us to statistically reject the hypothesis of perfect correlation between these constructs. Second, following the procedure recommended by (Anderson and Gerbing 1988), we performed chi-square difference tests as a more direct test. Constraining the latent correlation between ST and CA to 1.0 significantly worsened the model fit (Δχ^2^(1) = 8.15, *p* = 0.004). Similarly, constraining the correlation between ST and CB to 1.0 resulted in a highly significant degradation of fit (Δχ^2^(1) = 32.457, *p* < 0.001). These results provide strong statistical evidence that ST is distinct from both CA and CB.

**Table 3:** Discriminant validity.

**Item**	**EI**	**ST**	**CA**	**CB**	**FLE**
EI	**0.811**				
ST	0.520^***^	**0.748**			
CA	0.454^***^	0.764^***^	**0.898**		
CB	0.465^***^	0.797^***^	0.819^***^	**0.963**	
FLE	0.545^***^	0.666^***^	0.575^***^	0.641^***^	**0.742**

^***^p < 0.001. The bold values on the diagonal are the square roots of the average variance extracted (AVE).

Given the high correlations, we also assessed potential multicollinearity issues with CB as the dependent variable. The Variance Inflation Factor (VIF) results were all well below 10 (ST: 2.055; CA: 1.930; FLE: 1.579; EI: 1.378; Kutner et al., 2004), and the corresponding tolerance values all exceeded 0.1 (Menard, 2002). Although the minimum eigenvalue was 0.011, close to 0.01 (Hair et al., 2019), the collective evidence from these indicators suggests that multicollinearity is unlikely to substantially interfere with the parameter estimates of the model.

### 4.2 Descriptive statistics and correlation analysis

Table 4 presents the statistical characteristics of the core variables for 561 participants. As noted, scores for ST values were computed following the official intra-individual centering procedure (Schwartz, 2021) to correct for response style biases. This involves subtracting an individual's mean rating across all 21 value items (MRAT) from the raw value score. Thus, ST scores represent relative importance within an individual's value system and can yield negative means. Of particular note is the mean of the centered ST values was found to be negative. This indicates that, on average, the absolute importance of ST values for the sampled cohort of Chinese university students was slightly below the overall mean of the value scale used in this study. In contrast, the other variables (EI, FLE, CA, CB) were calculated as simple arithmetic means of their respective scale items, as is conventional for such scales. Correlation tests indicated that ST, EI, FLE, and CA were all significantly positively correlated with CB (*p* < 0.01).

**Table 4:** Descriptive statistics and zero-correlations among study variables.

**Item**	***M***	**SD**	**EI**	**FLE**	**ST**	**CA**	**CB**
EI	4.768	0.747	–				
FLE	5.182	1.080	0.462^**^	–			
ST	−0.182	0.440	0.432^**^	0.532^**^	–		
CA	5.768	1.113	0.413^**^	0.488^**^	0.670^**^	–	
CB	5.796	1.037	0.431^**^	0.536^**^	0.712^**^	0.793^**^	–

^**^p < 0.01.

### 4.3 Mediation effect test

**Figure 2:**
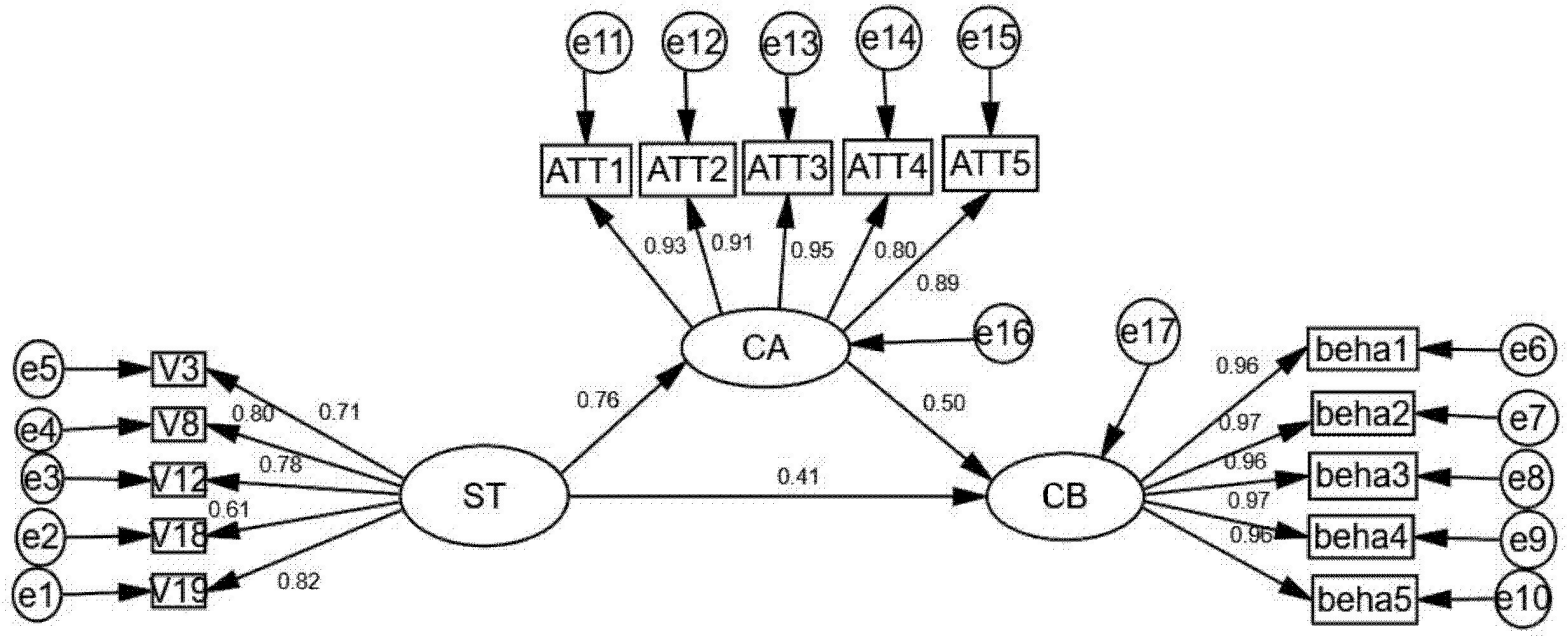
Parameters estimates of the general structural model.

To validate the mechanism through which ST values affects CB via CA, a mediation model was tested using AMOS 26.0 (see Figure 2). The model exhibited a good fit to the data (χ^2^/d*f* = 3.818, RMSEA = 0.071, SRMR = 0.030, CFI = 0.977, TLI = 0.972), meeting established standards (Kline, 2016). The model's explanatory power was assessed by examining the squared multiple correlations (*R*^2^) for the endogenous variables. ST explained 58.3% of the variance in CA (*R*^2^ = 0.583), while ST values and CA jointly explained 74.2% of the variance in CB (*R*^2^ = 0.742). According to Cohen's (1988) criteria, these *R*^2^^2^ values represent medium-to-large effect sizes, indicating that the model has substantial explanatory power for the endogenous latent variables.

The path coefficients and their significance levels are summarized in Table 5. All hypothesized paths were statistically significant, confirming the proposed mediation pathway. The Bootstrap mediation test results, presented in Table 6, indicated a significant indirect effect of ST on CB through CA (effect = 0.412, 95% CI [0.290, 0.542]), accounting for 48.2% of the total effect. To quantify the substantive impact of this mediation, we calculated the effect size *f*^2^ based on the *R*^2^ increase attributable to the mediator, which was 0.62, denoting a large effect according to Cohen's (1988) criteria.

**Table 5:** Scalar estimates of the research model.

**Path**	***B***	**S.E**	**C.R**	***p***	**β**
ST –> CA	0.904	0.049	18.504	^***^	0.764
CA –> CB	0.456	0.039	11.817	^***^	0.530
ST –> CB	0.443	0.048	9.138	^***^	0.413

^***^p < 0.001.

**Table 6:** Standardized direct, indirect, and total effects of the research model.

**Mediation Effects**			**Estimate**	**Bootstrap (95%)**		***p***
				**Lower**	**Upper**	
ind1	ST –> CA –> CB	*a*1 ^*^b1	0.412	0.290	0.542	^***^
Total		ind + *c*	0.856	0.780	0.944	^***^
*r*		ind/total	0.482	0.329	0.637	^***^

*a*1 ^*^ *b*1, indirect effect (product of path coefficient *a*1 and path coefficient *b*1); ind/total, indirect effect/total effect; ind + *c*, sum of indirect effect and direct effect; ^***^p < 0.001.

Furthermore, a *post-hoc* power analysis was conducted using G^*^Power 3.1. With an effect size *f*^2^ of 0.62 and a sample size of 561, the analysis demonstrated a statistical power of 1.00 for detecting the mediation effect, thereby substantially minimizing the risk of a Type II error and attesting to the robustness of the findings.

### 4.4 Moderating effect analysis of EI and FLE

The moderating effects were examined using the PROCESS macro (Model 7) to test a moderated mediation model. All continuous variables were standardized into *Z*-scores prior to analysis to facilitate the interpretation of regression coefficients. The independent variable and moderators, which were already standardized, were mean-centered using the macro's built-in function when creating the interaction terms to mitigate multicollinearity. The resulting moderation model explained 47.2% of the variance in CA when EI was included as the moderator, and 47.8% when FLE was the moderator.

**Figure 3:**
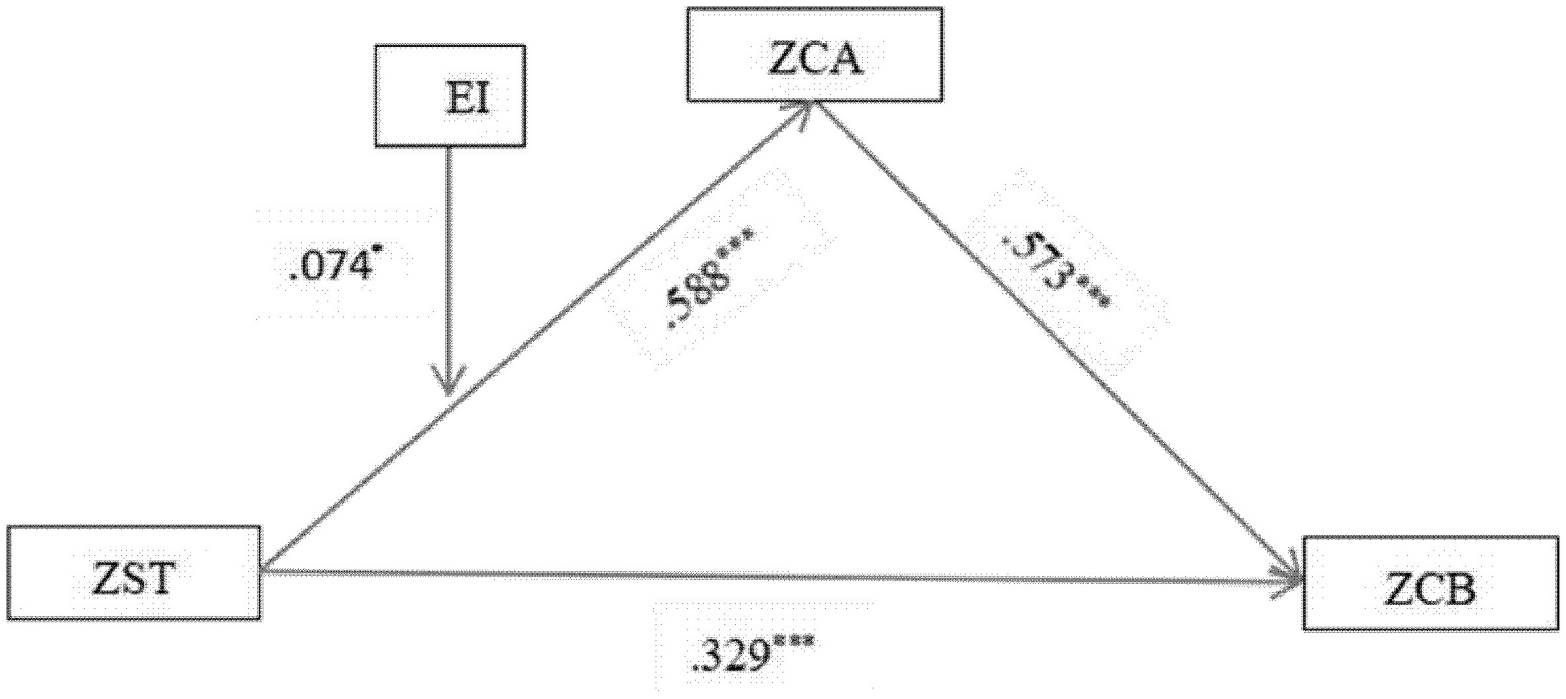
Standardized path coefficients and moderating effect (EI).

**Figure 4:**
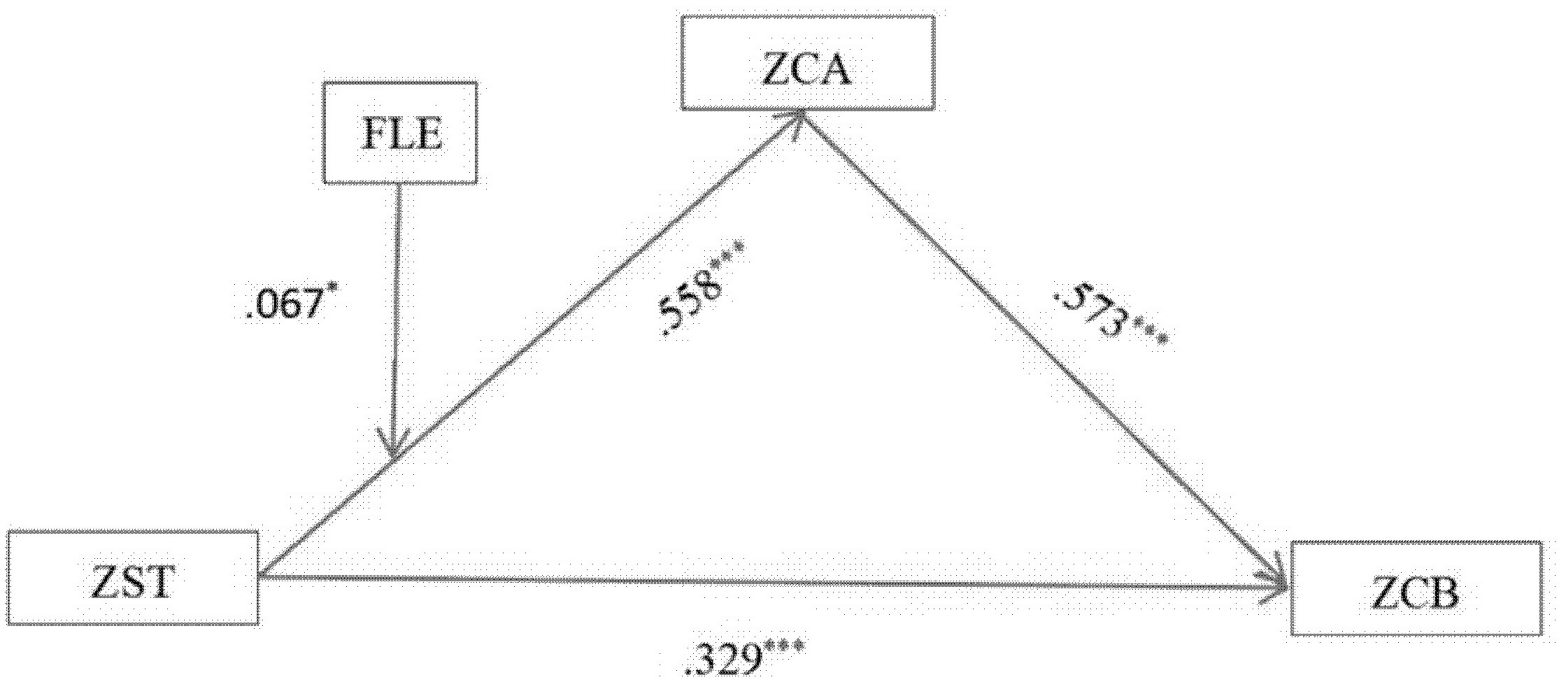
Standardized path coefficients and moderating effect (FLE).

**Figure 5:**
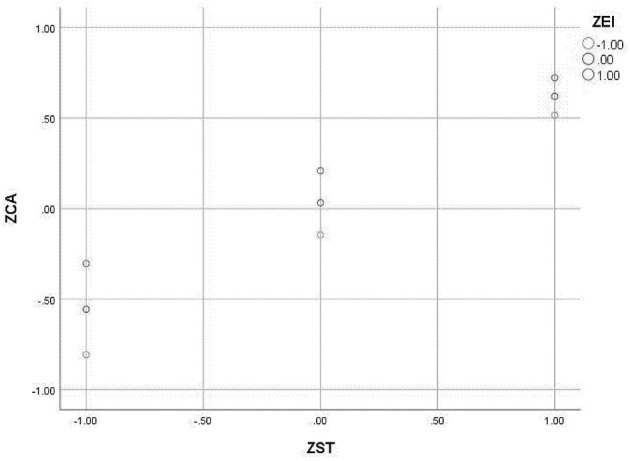
Scatter plot of ST, EI, and CA.

**Figure 6:**
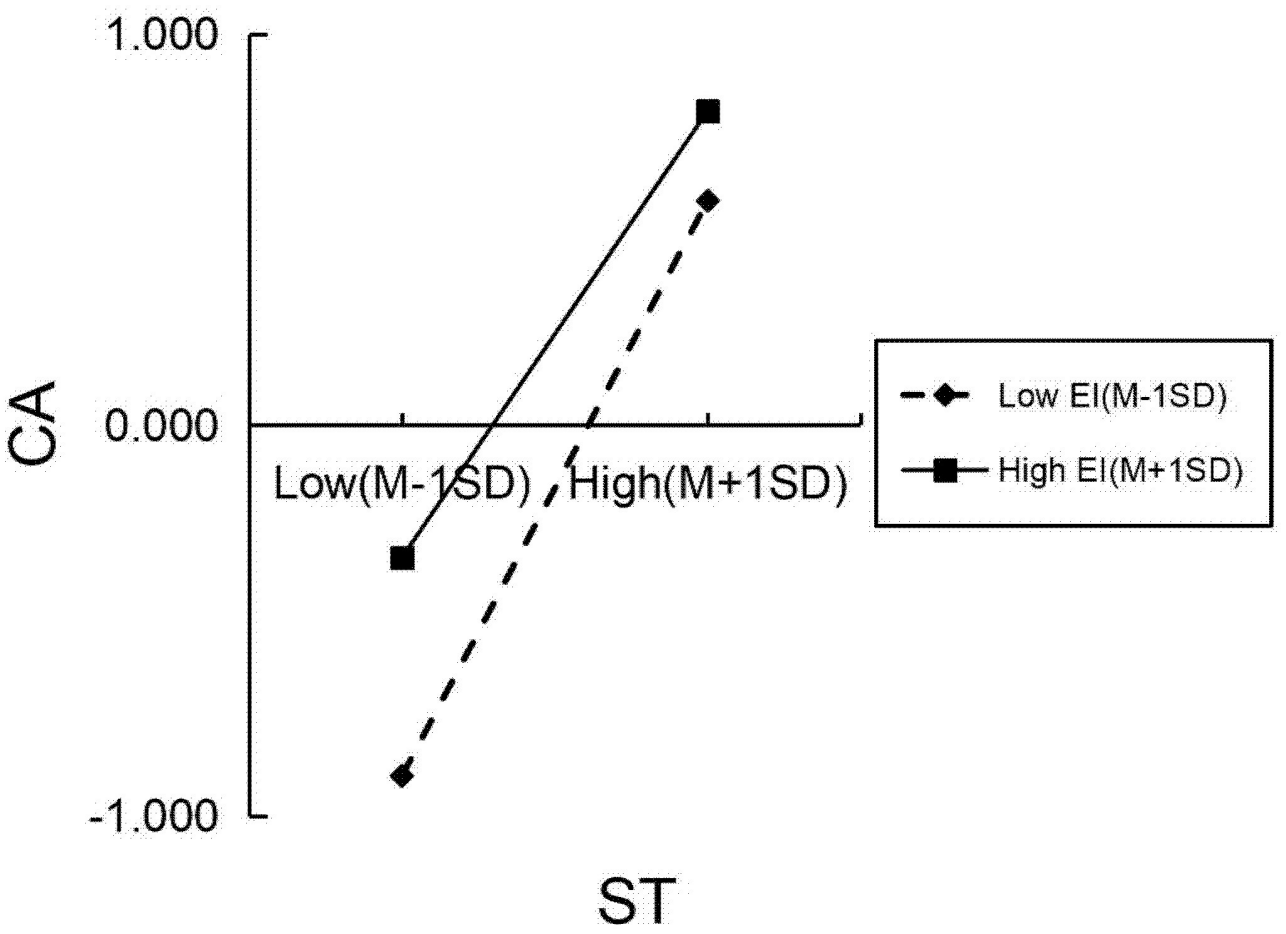
The pattern of moderation of EI on the slope between ST and CA.

Concerning the specific hypotheses, the interaction term EI × ST was a significant negative predictor of CA (β = −0.074, *p* < 0.05), thus supporting H6 (Table 7 and Figure 3). Similarly, the interaction term ST × FLE significantly and negatively predicted CA (β = −0.067, *p* < 0.05) (Table 8 and Figure 4). To further quantify the role of the moderators in the entire mediation mechanism, the index of moderated mediation was tested. The results showed that the index was significant for both EI (index = −0.042, 95% CI [−0.083, −0.008]) and FLE (index = −0.038, 95% CI [−0.068, −0.010]), confirming that both EI and FLE significantly moderated the indirect effect of ST values on CB via CA. The moderating effect of EI was first explored through a scatter plot (Figure 5), which displays the raw data points and illustrates the bivariate relationship between ST and CA at different levels of EI (−1 SD, Mean, +1 SD). To precisely quantify this interaction, a simple slopes analysis was conducted following the procedure recommended by Aiken and West (1991). The results are presented in a simple slopes plot (Figure 6), confirming that while ST values was a significant positive predictor of CA at both high and low levels of EI, the strength of this relationship was significantly weaker under high levels of EI (β = 0.514, *p* < 0.001) compared to low levels of EI (β = 0.662, *p* < 0.001). Furthermore, a Johnson–Neyman analysis was conducted to precisely identify the potential boundaries at which the moderating effect of EI becomes statistically significant or non-significant. Notably, no significance transition points were found within the observed range of the moderator EI. This indicates that the positive effect of ST values on CA remained statistically significant across the entire continuum of EI levels present in our sample. In other words, while the strength of the relationship between ST values and CA was attenuated under high EI (as shown by the conditional effects), ST values was a consistent positive predictor of CA regardless of whether a student's EI was low, average, or high.

**Table 7:** Regression results of moderated mediation of EI.

**Items**	**Parametric estimation**				**95% Confidence interval**		**Hypothesis testing**
	β	**SE**	***t***	***p***	**LLCI**	**ULCI**	
*Y* = ZCA constant	0.032	0.034	0.949	0.343	−0.034	0.098	
ZST	0.588	0.035	16.873	0.000	0.519	0.656	
ZEI	0.177	0.036	4.959	0.000	0.107	0.248	
*Y* = ZCB constant	0.000	0.024	0.000	1.000	−0.046	0.046	
ZST	0.329	0.032	10.327	0.000	0.266	0.391	
ZCA	0.573	0.032	18.010	0.000	0.511	0.636	
Int_1 = ZST × ZEI	−0.074	0.032	−2.334	0.020	−0.136	−0.012	H6^*^

^*^p < 0.05 (two-tailed).

**Table 8:** Regression results of moderated mediation of FLE.

**Items**	**Parametric estimation**				**95% confidence interval**		**Hypothesis testing**
	β	***SE***	***t***	***p***	***LLCI***	***ULCI***	
*Y* = ZCA constant	0.035	0.034	1.040	0.299	−0.031	0.102	
ZST	0.558	0.037	15.238	0.000	0.486	0.630	
ZFLE	0.192	0.036	5.297	0.000	0.121	0.263	
*Y* = ZCB constant	0.000	0.024	0.000	1.000	−046	0.046	
ZST	0.329	0.032	10.327	0.000	0.266	0.391	
ZCA	0.573	0.032	18.010	0.000	0.511	0.636	
Int_2 = ZST × ZFLE	−0.067	0.028	−2.397	0.017	−0.121	−0.012	H9^*^

^*^p < 0.05 (two-tailed).

**Figure 7:**
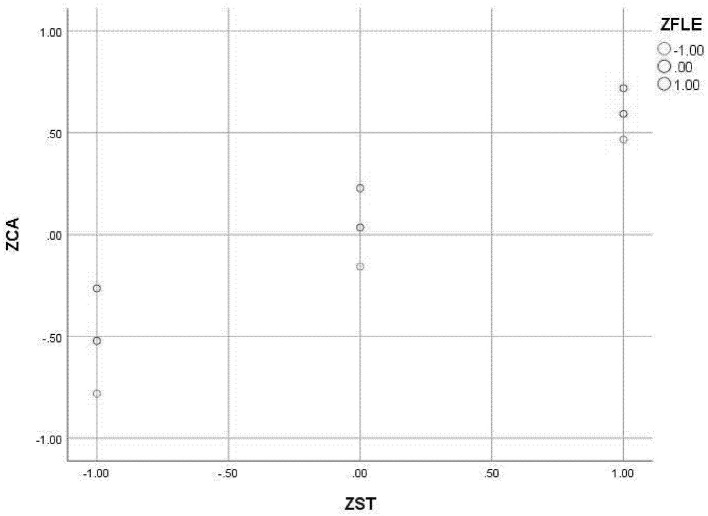
Scatter plot of ST, FEI, and CA.

**Figure 8:**
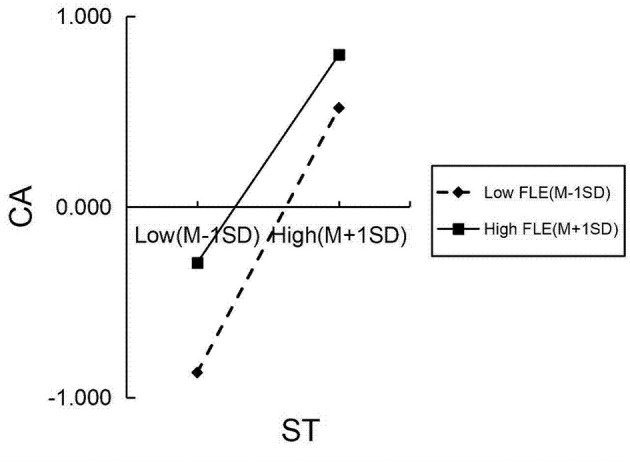
The pattern of moderation of FLE on the slope between ST and CA.

A parallel analytical approach was applied to FLE. The initial scatter plot (Figure 7) visualized the relationship between ST values and CA across different levels of FLE. Subsequent simple slopes analysis, depicted in Figure 8, yielded a highly similar pattern. The positive predictive effect of ST values on CA was significantly attenuated under high levels of FLE (β = 0.491, *p* < 0.001) relative to its effect under low levels of FLE (β = 0.624, *p* < 0.001). The application of the Johnson–Neyman technique specified that positive effect of ST on CA remained statistically significant across the entire continuum of EI levels present in our sample.

Furthermore, the conditional indirect effects of ST on CB via CA were examined at different levels of the moderators. The indirect effect remained significant across all levels, as indicated by bootstrap confidence intervals that excluded zero. However, its strength varied systematically. For EI, the effect was strongest at low levels (effect = 0.379, Boot SE = 0.045, 95% Boot CI [0.293, 0.471]), moderate at the mean (effect = 0.337, Boot SE = 0.040, 95% Boot CI [0.261, 0.419]), and weakest, yet still significant, at high levels (effect = 0.294, Boot SE = 0.044, 95% Boot CI [0.211, 0.385]). A highly similar pattern of attenuation was observed for FLE (Low: effect = 0.358, 95% Boot CI [0.274, 0.451]; Mean: effect = 0.320, 95% Boot CI [0.250, 0.399]; High: effect = 0.281, 95% Boot CI [0.212, 0.358]). This pattern provides robust quantitative support for the significant dampening effect of both EI and FLE on the mediation pathway.

A *post-hoc* power analysis was conducted using G^*^Power 3.1 to evaluate the statistical power for detecting moderating effects, based on the observed effect sizes (*f*^2^^2^), an alpha level of 0.05, a sample size of 561, and the number of predictors. The results showed that both moderating effects had small effect sizes (FLE: *f*^2^ = 0.0096, power = 0.47; EI: *f*^2^ = 0.0095, power = 0.46). Although the power values were below the conventional threshold of 0.80—which is primarily due to the small effect sizes—the interaction terms were still statistically significant (*p* < 0.05). This indicates that the large sample size enhanced the test's sensitivity, allowing for the detection of these subtle effects.

## 5 Discussion

### 5.1 ST values in a collectivist context

An interesting finding of this study is the negative mean of the centered ST values within our sample of Chinese university students. While this might appear counterintuitive given the emphasis on collectivism and harmony in Confucian-heritage cultures, this finding can be meaningfully interpreted through the lens of cultural psychology. The negative mean does not imply that ST values are unimportant. Rather, it suggests that in a cultural context where cooperative and prosocial behaviors are deeply ingrained as a social norm (Markus and Kitayama, 1991), the baseline level of ST-oriented motivation is universally high. When the prevailing cultural ethos already emphasizes collective welfare, the relative salience of ST values as a distinct motivator for any single individual may appear diminished when measured on a standardized scale. In addition, this finding resonates with prior research indicating that in collectivist nations, values related to conformity, security, and achievement (often associated with Conservation and Self-Enhancement) can be particularly emphasized, especially in contexts of rapid societal development (Feng, 2011; Liu et al., 2021). Our sample consists of young students navigating the intersection of traditional Confucian values and modern, competitive societal demands. In such an environment, values pertaining to academic success, future security, and fulfilling family expectations (which align more with Conservation and Self-Enhancement) might be acutely salient, potentially affecting the relative positioning of ST values when individuals evaluate their importance.

### 5.2 The dual mechanism of ST in CL

In this study, hypotheses H1, H2, and H3 were fully supported, demonstrating that ST values significantly predict CA and CB, with CA acting as a partial mediator. These findings align with and extend the Value–Attitude–Behavior Model (VAB Model) framework and Schwartz's theory of basic human values.

The mediation analysis revealed that the indirect effect of ST values on CB through CA accounts for 48.2% of the total effect. This indicates that nearly half of the influence of ST values on CB is channeled through the internal shift in attitudes. The substantial proportion of this mediation pathway highlights the critical role of cognitive and affective internalization (captured by CA) in translating abstract values into concrete actions within collaborative learning contexts. ST values explain 58.3% of the variance in CA, and together with the CA, they explain 74.2% of the variance in CB, suggesting that the ST-CA-CB model captures a dominant mechanism driving cooperative outcomes in English language classrooms. An effect size (*f*^2^) of 0.62 for the mediation effect further confirms its substantive impact.

This implies that interventions designed to enhance ST values are predicted to bring about meaningful and perceptible changes in how students cooperate. Pedagogical interventions aimed at promoting CB can be designed to target the specific pathways of ST. This entails: (1) nurturing benevolence by building caring relationships and a supportive classroom climate; and (2) integrating universalism by designing collaborative tasks that have authentic, shared goals aimed at a common good. The fact that the path from values to behaviors is partially, but not fully, mediated by attitude is equally important. It suggests that while attitude internalization is crucial, values can also influence behavior through more automatic or habitual pathways. This resonates with the cognitive restructuring mechanism whereby perceiving CL as a self-transcendent activity facilitates a shift in role identity from a competitive individual to a community member.

In conclusion, our findings provide robust empirical support for the cross-cultural applicability of Schwartz's theory in the specific context of English language education. They confirm that the VAB model is not only valid but is a potent framework for understanding student interactions in CL.

### 5.3 The moderating effect of EI and FLE

The hypothesized relationships and moderating effects involving EI and FLE were fully supported by the data (Figures 6, 8). Specifically, the results confirmed hypotheses H4 and H5, indicating a significant positive association of EI with both CA and CB. Similarly, hypotheses H7 and H8 were supported, demonstrating significant positive links between FLE and CA as well as CB. Crucially, the predicted negative moderating effects were also established: EI (H6) and FLE (H9) both significantly attenuated the positive relationship between ST values and CA. While the effect sizes of these interactions were small (EI: *f*^2^ = 0.0095; FLE: *f*^2^ = 0.0096), they were statistically significant and theoretically meaningful. The fact that these subtle yet systematic effects were detected within a model already explaining a substantial portion of the variance in CB (*R*^2^ = 0.742) underscores that they represent meaningful boundary conditions to the core VAB pathway.

The results confirmed that both EI and FLE served as significant positive predictors of CA and CB. These findings suggest that EI functions not only as a contributor to academic achievement but also as a core psychological capacity for regulating learners' affective and social responses in collaborative settings. Similarly, the significant positive influence of FLE on CA and CB reinforces existing literature that links enjoyment to increased willingness to communicate (Botes et al., 2022), highlighting how FLE stimulates positive emotions, strengthens learning motivation, and fosters participatory intentions in cooperative activities. Together, these results underscore the substantive roles of both emotional competence and positive emotional experience in facilitating CL processes.

The significant negative interaction between EI and ST values indicates that the positive influence of ST values on CA is attenuated under high EI. This result aligns with the Emotions as Social Information (EASI) theory proposed by Van Kleef (2009). According to the EASI framework, Individuals with high EI possess an enhanced capacity to perceive, use, understand, and manage emotions. In a cooperative context, this ability allows them to decode a rich array of real-time socio-emotional cues (e.g., group dynamics, nonverbal signals, task-specific demands) with remarkable proficiency. Consequently, for high-EI individuals, cooperative decisions are likely based more on a dynamic, situation-specific assessment informed by these immediate environmental cues rather than relying predominantly on a general, internal value like ST values. High EI, therefore, provides an alternative, skill-based pathway to cooperation. The reliance on a broad value becomes less critical when one possesses a sophisticated “socio-emotional tool kit” to navigate interactions effectively. For example, a high-EI student, detecting confusion in a peer during a group task, may cooperate by clarifying a point. This action is primarily triggered by the immediate socio-emotional cue, suggesting that for such individuals, situational awareness can temporarily supplant reliance on internal values like ST values when forming cooperative intentions.

Similarly, the negative interaction between FLE and ST values indicates that high enjoyment weakens the ST-CA link. This is best explained by a mechanism of motivational pathway competition. Students experiencing high FLE derive substantial intrinsic gratification and a sense of accomplishment from the language learning process itself. This positive emotional experience establishes a potent, self-sustaining “enjoyment-driven pathway” to cooperation. In this pathway, the motivation to cooperate stems directly from the inherent pleasure and engagement of the learning activity, rather than being primarily channeled through an abstract value system like ST values. For example, in a collaborative storytelling task, a student with high FLE derives immediate enjoyment from creating narratives and interacting with peers. Therefore, their cooperation is primarily driven by the intrinsic pleasure of the activity itself, rather than by the motivation of ST values.

In summary, the observed moderating effects demonstrate that the pathways within the Value–Attitude–Behavior Model are not merely additive but involve complex interactions where personal resources can attenuate the influence of core values. Both EI and FLE, as personal resources, modulate the ST-CA relationship by providing alternative, compelling routes to cooperation. High EI promotes a shift toward situationally-attuned cooperation based on real-time social intelligence, while high FLE promotes a shift toward enjoyment-driven cooperation based on intrinsic task engagement. This understanding enriches the VAB framework by highlighting the conditions under which value-attitude relationships can be moderated in non-intuitive ways, underscoring the multifaceted nature of motivation in cooperative language learning.

Consequently, the intricate interplay of values and personal resources outlined above provides clear guidance for designing instruction. Fostering cooperation in the language classroom can be achieved through multiple, and sometimes compensatory, pathways. For learners with lower levels of EI or FLE, who may be less adept at reading social cues or find the learning process less inherently enjoyable, the pathway via ST values is particularly crucial. For these students, explicitly cultivating a classroom culture centered on universalism and benevolence provides a stable, internalized compass for cooperation. Conversely, for students with higher EI or FLE, while ST values remain beneficial, their motivation to cooperate can be more effectively channeled through their distinct strengths. Instruction can leverage the social acuity of high-EI learners by assigning them roles that utilize their emotional skills, such as being a “group facilitator” responsible for managing group dynamics and ensuring all voices are heard. For high-FLE learners, designing collaborative tasks that are inherently engaging and enjoyable (e.g., game-based learning, creative projects) will tap directly into their pre-existing intrinsic motivation.

## 6 Study limitations

This study has several limitations. First, the sample was characterized by a high proportion of female participants and a dominance of students from language-related majors, which may limit the generalizability of the findings to male English learners and non-language major populations. Second, the analysis based on cross-sectional data only captures static associations, making it difficult to reveal the dynamic mechanisms underlying the internalization of values and the transformation of behaviors. Third, the exclusive use of a quantitative paradigm lacks qualitative exploration of deep motivations and contextual interactions in CL. Finally, from a methodological perspective, the reliance on composite scores for the moderation analysis, despite being a conventional approach, may not fully account for measurement error. Future studies could strengthen the evidence by employing latent variable modeling techniques, such as Latent Moderated Structural Equation Modeling (LMS), to further corroborate the moderating effects by directly controlling for measurement error.

## 7 Implications and future research directions

This study validates a moderated mediation model, clarifying how ST values influence CB through the mediating role of CA, while identifying the moderating effects of EI and FLE. These findings offer concrete implications for educational practice and establish a clear agenda for future research.

In terms of practical implications, educational practitioners should strategically employ blended cooperative learning to construct an “affective bridge” linking values, emotions, and behaviors. Instructional design should integrate scaffolded online tasks to enhance students' sense of control and self-efficacy, alongside high-interaction offline scenarios that activate the socio-emotional regulatory functions of EI. Adopting a blended “teacher + AI” feedback model can synergize timely technical input with personalized guidance, collectively boosting FLE and reinforcing self-efficacy. Furthermore, designing cooperative tasks that align with diagnosed student strategic preferences, particularly accounting for cultural differences in learning strategies, enables ST values to be translated into behaviors through cognitively congruent pathways.

Regarding the future research, efforts should focus on empirically examining the extended mediation chain “ST → FLE/EI → self-efficacy / independent learning strategies → CA → CB.” Future studies could manipulate instructional variables like feedback sources to test how they moderate key pathways. It is also crucial to initiate cross-cultural studies that sample diverse institutions and regions while controlling for course structure to isolate cultural and institutional effects. Employing longitudinal designs, multi-source data, and measurement invariance tests with multi-group SEM will be essential to circumvent common method bias, strengthen causal inference, clarify the model's generalizability, and enhance ecological validity. Research aligning with these directions will advance CL theory toward a multidimensional framework encompassing “design-strategy-culture.”

## Data Availability

The raw data supporting the conclusions of this article will be made available by the authors, without undue reservation.
